# Circulating miRNA-125b Is a Potential Biomarker Predicting Response to Rituximab in Rheumatoid Arthritis

**DOI:** 10.1155/2014/342524

**Published:** 2014-03-20

**Authors:** Isabelle Duroux-Richard, Yves-Marie Pers, Sylvie Fabre, Meryem Ammari, Dominique Baeten, Guillaume Cartron, Isabelle Touitou, Christian Jorgensen, Florence Apparailly

**Affiliations:** ^1^Inserm U844, CHU Saint Eloi, Bâtiment INM, 80 Avenue Augustin Fliche, 34295 Montpellier Cedex 5, France; ^2^Université Montpellier I, UFR de Médecine, Boulevard Henri IV, 34090 Montpellier, France; ^3^Clinical Department for Osteoarticular Diseases, CHU Lapeyronie, Avenue Gaston Giraud, 34295 Montpellier, France; ^4^Clinical Immunology and Rheumatology, Academic Medical Center/University of Amsterdam, 1105 AZ Amsterdam, The Netherlands; ^5^Département d'Hématologie, CHU Saint Eloi, 80 Avenue Augustin Fliche, 34295 Montpellier, France; ^6^UMR-CNRS 5235, Université Montpellier II, Place Eugène Bataillon, 34095 Montpellier, France; ^7^Unité des Maladies Autoinflammatoires, Laboratoire de Génétique, CHU Lapeyronie, Avenue Gaston Giraud, 34295 Montpellier, France

## Abstract

Although biologic therapies have changed the course of rheumatoid arthritis (RA), today's major challenge remains to identify biomarkers to target treatments to selected patient groups. Circulating micro(mi)RNAs represent a novel class of molecular biomarkers whose expression is altered in RA. Our study aimed at quantifying miR-125b in blood and serum samples from RA patients, comparing healthy controls and patients with other forms of rheumatic diseases and arthritis, and evaluating its predictive value as biomarker for response to rituximab. Detectable levels of miR-125b were measured in total blood and serum samples and were significantly elevated in RA patients compared to osteoarthritic and healthy donors. The increase was however also found in patients with other forms of chronic inflammatory arthritis. Importantly, high serum levels of miR-125b at disease flare were associated with good clinical response to treatment with rituximab three months later (*P* = 0.002). This predictive value was not limited to RA as it was also found in patients with B lymphomas. Our results identify circulating miR-125b as a novel miRNA over expressed in RA and suggest that serum level of miR-125b is potential predictive biomarker of response to rituximab treatment.

## 1. Introduction

Rheumatoid arthritis (RA) is a chronic, systemic inflammatory autoimmune disorder that may affect many tissues and organs but principally attacks the joints. RA is a multifactorial disease of unknown aetiology and complex pathogenesis, consisting of at least two subtypes, with different causes and severity [1, 2]. The clinical course of RA fluctuates and prognosis is unpredictable. A major issue is that up to 70% of patients with recent onset of RA show evidence of radiological erosions within 3 years. In the long term, major outcomes include joint deformity and misalignment, need for joint replacement surgery, functional disability, and premature death due to accelerated atherosclerotic cardiovascular and coronary heart diseases [3]. Numerous studies have demonstrated that aggressive treatment of early RA results in better clinical outcome [4]. Nine biologics are available for RA treatment, often used in combination with methotrexate. Each type targets a specific inflammatory mechanism and has largely improved the outcome of RA in many patients [5, 6]. Biologics are however prescribed on a trial-and-error basis when methotrexate alone has failed, response is heterogeneous, and roughly one-third of patients are nonresponders. It may thus take some time to find the best drug for a patient. Considering the high cost of biologics and the possibility of severe side effects, the identification of predictors of response to biologic therapies would improve patient care and medical cost-effectiveness.

Clinical and serological characteristics solely are insufficient to predict treatment outcome. Anticitrulline peptide antibodies (ACPA), elevated CRP, serum levels of EGF, MCP-1 or TNFR, and gene profiling have been proposed to identify responders to biologics [7–9]. More recently, micro(mi)RNAs have emerged as a new category of biomarkers and patients with RA have clear alterations of the expression of miRNAs [10]. Conserved throughout evolution, miRNAs are an abundant class of endogenous, short noncoding, regulatory RNA molecules that control gene expression in a sequence-specific manner by targeting mRNAs for degradation or translational repression. The potential value of miRNAs as molecular biomarkers for diagnosis, prediction of disease outcome, and prediction of therapeutic response is well documented in cancer [11], whereas in RA it remains poorly explored [12, 13]. Since 2008, the presence of miRNAs in human body fluids has been documented, and several studies reported the optimization of direct miRNAs detection in blood or sera [14, 15]. Recently, Murata et al. identified a signature of seven plasma miRNAs as diagnostic biomarkers specific for RA patients, even ACPA-negative [16]. Nevertheless, there are no reports so far about miRNA expression predicting treatment outcome in patients with RA.

Rituximab is the world's best-selling cancer drug and was originally developed to treat non-Hodgkin's lymphoma [17]. It is a chimeric monoclonal antibody directed against the CD20 surface antigen of B cells and FDA approved in 1997 to be used in combination with methotrexate to treat RA patients who have moderate-to-severe active disease and have failed one or more anti-TNF drugs [18]. Large randomized controlled trial has demonstrated efficacy in longstanding RA patients who failed to respond to methotrexate or anti-TNF drugs [19]. Despite effective depletion of circulating B cells in nearly all patients [20] and complete resolution of inflammation in some cases, only half of them however respond to rituximab treatment. Consequently, there is much interest in identifying molecular biomarkers that predict whether a patient will respond or not to rituximab. In addition to sharing a common treatment, RA and a substantial fraction of lymphomas share pathogenic inflammatory responses due to aberrant activation of NF-*κ*B signals [21]. Since miR-125b is an evolutionary conserved miRNA that regulates signal pathways of inflammation [22], B cell differentiation [23, 24], TNF production, and apoptosis [25] that are biological pathways of importance for both lymphoma and RA, we assessed whether miR-125b is deregulated in RA and useful as potential biomarker predictive for rituximab response.

## 2. Materials and Methods

### 2.1. Patients and Healthy Controls

Fresh peripheral blood and serum samples were obtained from healthy donors (*n* = 13) with no history of autoimmune diseases or patients with osteoarthritis (OA, *n* = 7) and rheumatoid arthritis (RA, *n* = 48) fulfilling the 2010 ACR/EULAR criteria [26]. Among the 48 RA patients, we included 32 patients treated by rituximab. Samples were also obtained from patients with receptor-associated periodic syndrome (TRAPS, *n* = 5) and spondyloarthropathies (SpA, *n* = 15). Informed consents were provided in accordance with procedures approved by the local human ethics committee (Comité de Protection des Personnes Sud Méditerrannée IV: ID RCB 2008-A01087-48).

The characteristics of RA patients are summarized in Table 1. Patients were assessed for overall disease activity using the 28-joint-count Disease Activity Score (DAS28) as previously described [27]. The criteria for patient eligibility were combined methotrexate (MTX) treatment; DAS28 ≥ 4.5; and resistance to at least 2 Disease Modifying Antirheumatic Drugs (DMARDs) (MTX and anti-TNF included). For one month or more before the start of this study, every patient was given stable doses of oral corticosteroids and did not receive any intra-articular steroid injections. Patients were treated with rituximab (MabThera, Roche) as recommended by the manufacturer and the French Drug Agency ANSM (intravenously 1,000 mg one time at day 0 and day 15). RA patients were separated in two subgroups according to their clinical response to the rituximab after 3 months (M3) of treatment (DAS28 M3-M0), following the EULAR criteria: for nonresponders (NR), DAS28 > 5.1 and the ratio DAS28 M3-M0 ≤ 0.6; for good and intermediate responders (R), DAS28 < 3.2 or DAS28 > 3.2 and DAS28 M3-M0 > 1.2.

Staging procedures for lymphoma patients (*n* = 13) were in accordance with international recommendations [28]. Clinical characteristics are summarized in Table 2. None of these patients presented concurrent RA. Patients were treated either by rituximab alone for four weekly infusions or by rituximab-chemotherapy regimen when appropriate. Response was assessed 4–6 weeks later according to international recommendations [28, 29]. Patients in complete or partial response were classified as responder patients and those in stable disease or progressive disease were classified as nonresponder patients.

### 2.2. Blood RNA Isolation and miRNA Quantification Using RT-qPCR

Blood samples were collected using EDTA-coated tubes (BD Vacutainer 5 mL; BD Diagnostics, France) according to standard procedure. Aliquots of 0.5 mL of blood samples were immediately transferred to 1.2 mL of RNA later medium (Applied Biosystems) and stored at −20°C. Total RNA was extracted using a modified protocol from the Ribopure-Blood RNA isolation kit (Applied Biosystems). Briefly, 10 *μ*L glacial acid (Sigma, France) was added to blood cell lysate (800 *μ*L, steps 1 and 2 according to the manufacturer's instruction). The samples were extracted with acid phenol/chloroform, 1 mL of GuSCN lysis solution (4 M guanidinium thiocyanate, 25 mM sodium citrate, 0.5% (w/v) sodium N-lauroyl sarcosinate), and 0.1 M beta-mercaptoethanol and 1.25 volumes of ethanol were added to the aqueous phase. The samples were passed through a filter cartridge and washed, first with wash solution 1 (70% EtOH/30% GuSCN lysis solution) and second with wash solution 2 (80% EtOH/50 mM NaCl). The RNA was eluted in 100 *μ*L elution solution preheated to 80°C and stored at −20°C. The concentration and integrity of RNA were determined by NanoDrop ND-1000 spectrophotometry (NanoDrop Tech, Rockland, Del) and by a Bioanalyser Agilent 1.

For miRNAs analysis, 10 ng of total RNA was reverse transcribed using 50 nM human microRNA specific stem-loop RT primers, 50 units/*μ*L MultiScribe reverse transcriptase, 10XRT buffer, 100 mM each dNTPs, and 20 units/*μ*L RNase inhibitor (Applied Biosystems). Reaction mixtures (15 *μ*L) were incubated in a thermocycler Mastercycler (Eppendorf, France) for 30 minutes at 16°C, 30 minutes at 42°C, and 5 minutes at 85°C and then maintained at 4°C. Real-time PCR was performed on the resulting complementary DNA using TaqMan microRNA specific primers and TaqMan Universal PCR Master Mix. All the experiments were performed according to the manufacturer's protocols, using a pipetting robotic platform epMotion 5070 (Eppendorf) and a LightCycler 480 Detection system (Roche, France). The expression of the U6B small nuclear RNA (RNU6B) was used as endogenous control for data normalization. Relative expression was calculated using the comparative threshold cycle (Ct) method.

### 2.3. MicroRNA Extraction from Serum and Quantification Using RT-qPCR

Whole blood was separated into serum and cellular fractions within 2 h following collection. Sera were stored at −20°C. RNA extraction of 400 *μ*L serum was performed by acid phenol:chloroform extraction and precipitated with ethanol over night at −20°C [14]. After precipitation, 40 *μ*L of sterile water was added to the RNA isolation.

Typically, a 15 *μ*L reverse transcriptase reaction contained 6.7 *μ*L of purified RNA and reverse transcription was performed according to the manufacturer's instruction. Real-time PCR was performed on the resulting complementary DNA using TaqMan microRNA specific primers and TaqMan Universal PCR Master Mix. Since U6 and 5S rRNA were degraded in serum samples [14, 15], results were normalized by subtracting the global miRNA levels in the sample (average Ct of 6 miRNAs, hsa-miR-142-3p (ID 000464), hsa-miR-142-5p (ID 002248), hsa-miR-24 (ID 000402), hsa-miR-181d (ID 001099), hsa-miR-15b (ID 000390), and hsa-miR-125b (ID 000449) for RA sera; average Ct of 4 miRNAs, hsa-miR-16 (ID 000391), hsa-miR-24, Let7-a (ID 000377), and hsa-miR-125b for B lymphoma sera) from the level Ct of miR-125b.

### 2.4. Statistical Analysis

Patients' parameters were analyzed with the nonparametric Wilcoxon signed-rank test. Correlations with miR-125b expression levels were quantified with the Spearman's correlation test and the Fisher transformation was applied. All other data were analysed statistically using the Mann-Whitney *U* test. *P* values less than 0.05 were considered statistically significant. The Power and Precision V3 Software (http://www.power-analysis.com/) was used to calculate the 1-*β* error (the probability of a *P* = 2*α* < 0.01 not appearing at random) for the difference in sera levels of mir-125b between responders and nonresponders.

## 3. Results

### 3.1. Quantification of Mature miR-125b in RA Blood Samples

**Figure 1:**
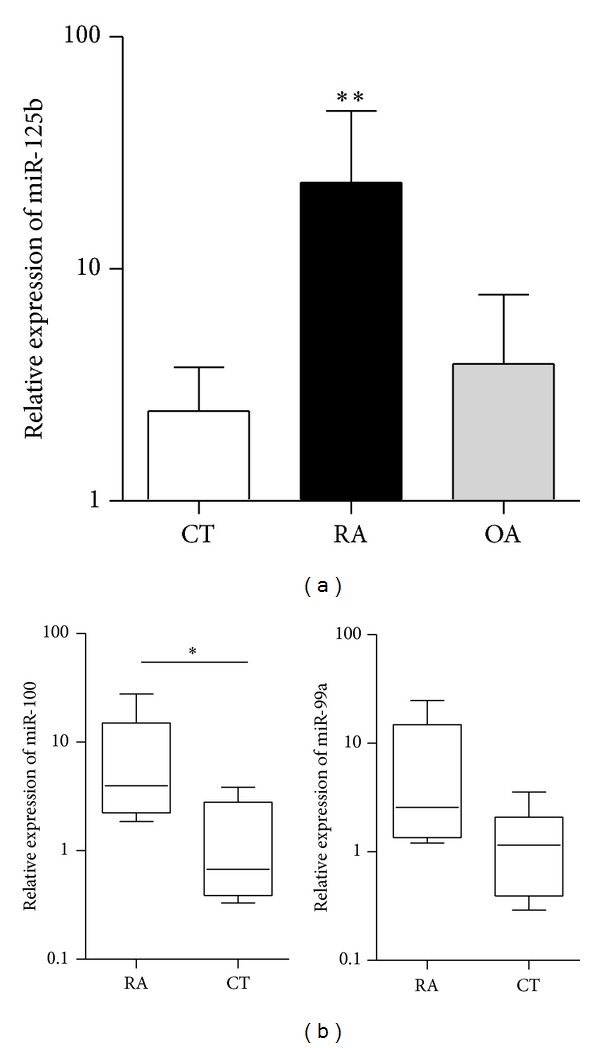
miR-125b is overexpressed in RA blood. (a) Expression levels of miR-125b in blood of healthy individuals (CT, *n* = 13) and patients with osteoarthritis (OA, *n* = 5) and rheumatoid arthritis (RA, *n* = 16). (b) Expression levels of miR-100 and miR-99a in blood samples from CT (*n* = 7) and RA patients (*n* = 6). *P < 0.05, **P < 0.01 as determined by Mann-Whitney test.

Using microarray technology, we identified miR-125b as deregulated in pooled blood samples from RA patients as compared with healthy donors (unpublished data). First, we validated these data analysing miR-125b expression on individual blood samples collected from sixteen RA patients having severe disease and similar clinical features (Table 1). Total RNAs were isolated from 0.5 mL of whole blood and the mature miR-125b form was quantified using RT-qPCR (Figure 1(a)). Expression levels were normalized with respect to U6 gene expression and expressed as 2^−ΔCt^. Mature miR-125b relative expression was significantly higher in patients with full-blown RA than in samples from healthy donors and OA patients (*P* = 0.026). More specifically, we observed that miR-125b was overexpressed in 12 of 16 (75%) blood samples (median of healthy donors = 2.22).

In humans, there are two paralogs coding for the same mature miR-125b sequence. They are located on two different polycistronic miRNA clusters on chromosomes 11 (hsa-miR-125b-1) and 21 (hsa-miR-125b-2), harbouring miR-100/let-7a-2/miR-125b-1 and miR-99a/let-7c/miR-125b-2, respectively [30]. To determine whether the increased mature miR-125b expression observed could be preferentially related to the upregulation of one of these 2 miRNA clusters, we quantified one miRNA encoded by each cluster (Figure 1(b)). Although only miR-100 reached statistical significance, both miR-99 and miR-100 were similarly overexpressed in blood from RA patients as compared with healthy donors, suggesting that both clusters are similarly deregulated in RA.

### 3.2. Mature miR-125b Overexpression in Serum Samples from RA Patients

**Figure 2:**
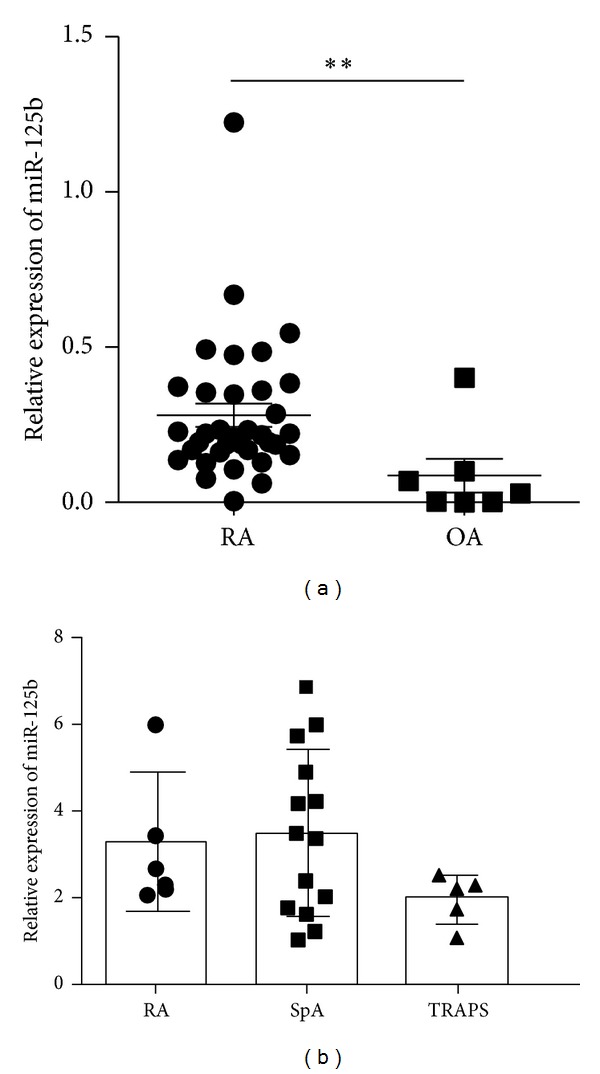
Serum levels of miR-125b in various rheumatic diseases. (a) Serum levels of mature miR-125b in patients with rheumatoid arthritis (RA, *n* = 32) or osteoarthritis (OA, *n* = 7). (b) Serum levels of miR-125b in patients with tumor necrosis factor receptor periodic syndrome (TRAPS), spondyloarthropathies (SpA), and rheumatoid arthritis (RA). miR-125b was quantified by RT-qPCR as described in M&M (*n* = 5–15/group). **P < 0.01 as determined by Mann-Whitney test.

Since miRNAs are also present in serum, we investigated whether miR-125b upregulation could also be measured in RA serum samples. Using real-time quantitative PCR [24, 31], detection of miR-125b was confirmed with serum from 32 patients with full-blown RA (Figure 2(a)). In addition, to determine whether this miRNA is specific for RA, its expression level was analysed in patients with OA. Analyses showed that serum miR-125b expression levels were significantly different between RA and OA patients (*P* < 0.01). To assess the potential of serum miR-125b as noninvasive biomarker of RA, serum samples from other rheumatic diseases including TRAPS and SpA were also analysed (Figure 2(b)). The expression levels of miR-125b measured in serum from RA patients were not different from other rheumatic disorders tested. Although further studies will be necessary, our data suggested that change in serum miR-125b is not specific for RA.

### 3.3. High Expression of miR-125b in RA Serum Predicts Good Response to Rituximab Therapy

**Figure 3:**
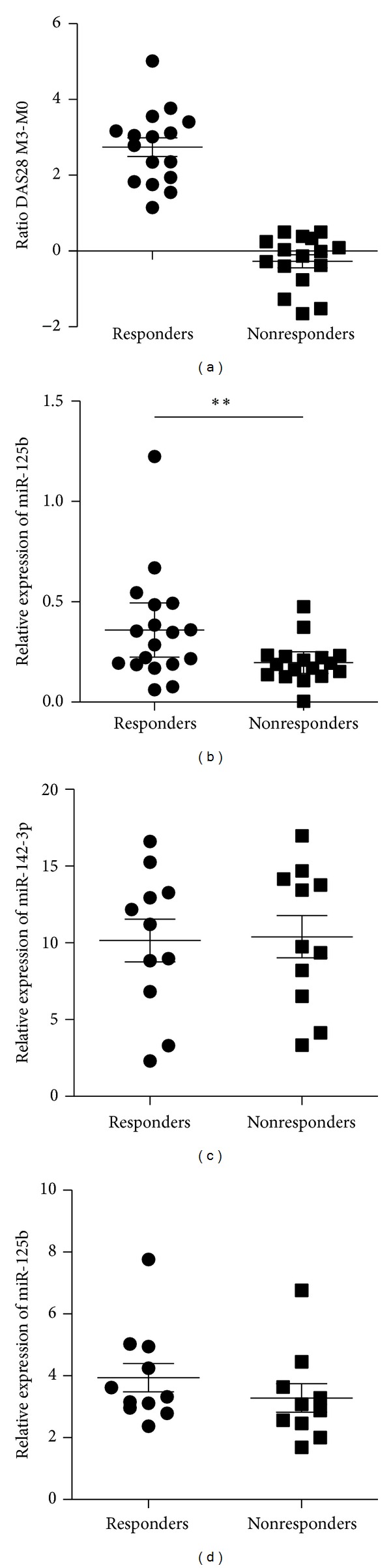
miR-125b as predictive biomarker for rituximab treatment outcome in RA. (a) DAS28 M3-M0 represents the difference at baseline versus 3 months after rituximab treatment. Patients were considered nonresponders (NR, *n* = 16) or responders (R, *n* = 16) according to EULAR criteria. Serum mature miR-125b (b), miR-142-3p (c), and miR-142-5p (d) are quantified in RA patients according to their clinical response. *P < 0.05, **P < 0.01 as determined by Mann-Whitney test.

We next determined whether the detection of miR-125b in serum of patients with active RA could be used as biomarker to predict clinical responses to rituximab (Figure 3). Serum samples were collected prior to treatment and miR-125b expression levels quantified by RT-qPCR. When the 32 RA patients were divided in two sub-groups according to their clinical response to rituximab after 3 months of treatment (Figure 3(a), *P* < 0.001), results showed that high expression of miR-125b was associated with a good response to anti-CD20 therapy (*P* = 0.002, Figure 3(b)). Indeed, serum levels of miR-125b before the initiation of treatment were higher in good responders compared with nonresponders, while two other miRNAs also detectable in serum, namely, miR-142-3p and miR-142-5p, were not expressed at significantly different levels in both groups of patients (Figures 3(c) and 3(d)). These data suggest that RA patients with low expression of miR-125b at the time of disease flare have significantly lower chance to improve clinically after 3 months of rituximab treatment and that serum abundance of miR-125b could be used as predictive biomarker. With mean value 0.36 ± 0.26 for responders (*n* = 16) and 0.19 ± 0.12 for nonresponders (*n* = 16), the power analysis yielded a 1-*β* value of 71%. Power calculations estimated that, keeping the difference between means and the SD-values constant, 40 patients in each treatment group would be the minimum sample size required so that 1-*β* value will be close to 100%. This signifies that an analysis of a single sera sample for mature miR-125b contents will serve as a very good predictor or clinical marker for a patient's response to treatment.

### 3.4. Predictive Value of miR-125b in the Serum of Patients with B Lymphoma under Rituximab Treatment

**Figure 4:**
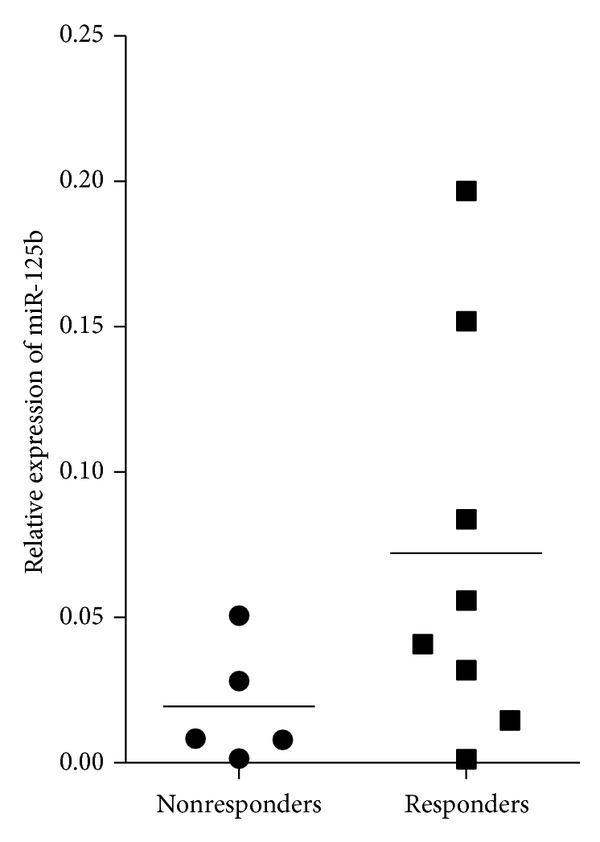
Serum miR-125b predicts rituximab response in B lymphoma patients. Expression levels of miR-125b were quantified by RT-qPCR as described in M&M in the serum of patients with B lymphoma before initiation of rituximab treatment. Clinical response was analyzed 4–6 weeks later and patients were considered nonresponders (NR, *n* = 5) or responders (R, *n* = 8) according to international recommendations.

To assess whether high serum expression levels of miR-125b could predict the therapeutic outcome for disorders other than RA treated with rituximab, we measured its expression levels in the serum of thirteen patients with B lymphomas (Figure 4). Samples were collected before initiation of the rituximab treatment and patients were divided into responders versus nonresponders according to their clinical response. Although it did not reach statistical significance, there was a tendency of higher expression levels of miR-125b in the group of responders to rituximab versus nonresponders. Consistent with results in RA samples, miR-125b was overexpressed in 6 out of 8 (75%) serum samples (median of nonresponders = 0.01) from responder patients, suggesting that serum concentration of miR-125b is a potential biomarker of rituximab response, predicting treatment outcome for patients with RA and B lymphomas.

## 4. Discussion

Rheumatoid arthritis (RA) is a heterogeneous disorder with fluctuating and unpredictable clinical course. Although a large panel of therapies is available to clinicians, they sometimes fail or produce partial responses, rarely achieve sustained remission, and are associated with systemic complications. Most importantly, prolonged delay in achieving adequate disease control impacts quality of life for RA patients. Current classification of patients based on the clinical phenotype and autoantibody production is not optimal and today's main challenge is to treat RA patients as early as possible with the most adequate treatment. Towards this goal, identification of biomarkers enabling to match therapies with specific subgroups of patients is of major interest. Recently, miRNAs emerged as an important class of new blood-based biomarkers that can associate their specific expression profile with disease development and severity, as well as response to treatment. This is particularly well documented across a large spectrum of cancers [32]. The possibility to detect miRNAs, not only in diseased tissues, but also in body fluids, opened great opportunities for these molecules in terms of clinical application [14, 15, 31, 33, 34]. As few publications suggested that miRNAs could be used as biomarkers with diagnosis implications in RA [16, 35], we thought to investigate whether miRNAs could also predict response to therapy. We found that mature miR-125b is overexpressed in both serum and blood samples from RA patients and well differentiated them from healthy donors or patients with OA. It is however not specific for RA as we also found elevated miR-125b levels in samples from patients with other rheumatic diseases including TRAPS and SpA. Furthermore, high expression levels of circulating miR-125b before initiation of treatment with rituximab were associated with good clinical response.

Although circulating miRNA still remains a new field in RA, one publication compared the quantification of 5 miRNAs in the plasma and synovial tissue of RA patients and shows that miRNAs released in the synovial fluid are similar to synovial tissue miRNAs, but distinct from plasma miRNAs [13]. The authors conclude that the detection of cell-free miRNAs in the serum of RA patients is more likely reflecting distinct composition and activation status of the haematopoietic compartment than of the joint space; suggesting that the systemic inflammatory aspect of the disease more than the rheumatic part might predominantly influence the blood miRNA pattern in RA. This might explain why we found that miR-125b well differentiated RA patients from OA individuals but not from patients with rheumatic disorders displaying a systemic inflammatory component such as TRAPS and SpA. Moreover, this is in agreement with the literature as miR-125b belongs to the miRNAs that are involved in haematopoiesis. It is highly expressed in normal haematopoietic stem cells (HSC) and is progressively down-regulated in committed myeloid and lymphoid progenitors. Its abnormal overexpression in these populations is associated with the development of lymphoproliferative diseases [36], myelodysplasia, and acute myeloid leukemia or B-cell acute lymphoid leukemia [23, 37, 38]. More recently, miR-125b overexpression has been correlated with the maintenance of the naive state of CD4^+^ T cell, preventing CD4^+^ T cell differentiation and acquisition of an effector/memory phenotype by CD4^+^ T cells [39]. Interestingly, when analyzing the data published by Li and colleagues, miR-125b expression appears significantly up-regulated in CD4^+^ T cells of RA patients compared with healthy controls [40]. Finally, very high miR-125b levels have been proposed to inhibit early steps of differentiation induction of granulopoiesis [41]. Overall, these data suggest that high levels of miR-125b in the blood of RA patients might reflect defective lineage differentiation and enhanced blast proliferation, leading to abnormal abundance of haematopoietic progenitors blocked at early stage of their lineage differentiation program, similar to what is observed in leukemic malignancies.

A contribution from B lymphocyte-derived miR-125b was also possible as miR-125b is up-regulated in germinal center (GC) lymphocytes compared to memory B cells [24]. Finally, B cells play a critical role in the pathogenesis of RA as B-cell depletion shows positive results for the treatment of RA. However, the expression levels of miR-125b in RA serum were not altered after 3 months of rituximab treatment (data not shown), further suggesting that the over-expression of miR-125b in RA patients is more likely due to the contribution of the T lymphocyte and myeloid compartments than of the B lymphocytes. The detection of miRNAs in serum was quite unexpected as RNA molecules are unstable in the circulation. Studies showed that extracellular miRNAs exhibit high stability in body fluids as they circulate associated with proteins or within membrane vesicles such as exosomes or microparticles [15, 33]. In addition to the usefulness of circulating miRNAs as biomarkers, there are evidences regarding their possible function in distant cell-to-cell communication. Identification of the form and source of extracellular miR-125b will thus clarify its role in arthritis.

Although blood is easily accessible, noninvasive and of great interest for new biomarker discovery, very few studies report the detection of miRNAs in plasma or serum of RA patients [12, 31, 42]. Until now, only Murata and coworkers suggested miRNAs as potential biomarkers in RA [13, 16]. Authors showed that plasma miR-132 concentrations were significantly lower in RA than in healthy donors and that plasma levels of miR-16 were correlated with disease activity assessed by the DAS28 (28-joint Disease Activity Score), although not specific for RA since similarly altered in the plasma from OA patients. More recently, they identified a signature of seven miRNAs termed ePRAM (for “estimated probability of RA by plasma miRNAs”), elevated in RA plasma relative to healthy donors, and allowing RA diagnosis with high specificity and sensitivity, even in ACPA-negative patients [16]. Interestingly, the ePRAM signature includes miR-125a-5p that belongs to the miR-125 family, consisting of three homologs in humans (hsa-miR-125a, hsa-miR-125b-1 and hsa-miR-125b-2), which are transcribed from three different loci that code for different mature sequences with the same seed region and therefore might have similar functions [43]. In the present study, we show that miR-125b is also detectable, both in blood and serum samples, and significantly elevated in RA patients as compared to healthy controls and OA patients, extending the observations of Murata and colleagues to the miR-125 family members as potential biomarkers in RA. However, we did not find significant correlation with DAS28, HAQ and CRP (data not shown). Circulating miR-125b has been reported as part of the blood-based miRNA signatures of ovarian and prostate cancers [15, 34]. Here, we add miR-125b to the short list of blood-based miRNAs as biomarkers in RA, and more importantly show for the first time that miR-125b can possibly predict disease biotherapy success in RA.

Previous reports in the cancer field have indicated that miRNAs could play an important role in predicting drug responses, but nothing was known until now in RA. Molecular prediction of treatment response for RA patients is still unmet medical need and is particularly important to help rheumatologists to select the optimal therapy for a given patient as they benefit from a large panel of biological drugs for which the benefice/risk ratio is not equal depending on patients and disease duration [44]. In case of rituximab, better clinical response was found associated with lower levels of IFN-*γ* and B-cell activating factor (BAFF), with the Fc*γ* receptor III (Fc*γ*RIII) genotype and the C/G-174 polymorphism in interleukin-6 (IL-6) gene [45]. In addition, an initial nonresponse to rituximab depends on circulating preplasma cell numbers at baseline and on incomplete depletion following treatment. Recently, a prospective study showed that good clinical response to rituximab is associated with the presence of B cell markers in the serum, more specifically with rheumatoid factor positivity or high anti-CCP antibody positivity and elevated IgG levels [46]. However, we found no correlation between the presence and/or levels of anti-CCP antibodies and miR-125b expression levels (data not shown). Our data suggest that miR-125b might be considered as an additional predictive biomarker for response to rituximab treatment as its expression level in the circulation, before the initiation of rituximab treatment, predicts therapy outcome. Importantly, this was not only observed for patients with RA but we found similar tendency for patients with B lymphoma, suggesting a broader application. Indeed, further determination is required including reproducibility experiment using other cohorts and comparison with other control groups such as patients with infection. In addition, RA patients used in our study have established disease and failure to anti-TNF drugs. It is thus not clear yet whether miR-125b can predict response to rituximab for patients with earlier RA and who had never received any biological therapy.

## 5. Conclusions

In conclusion, we have identified miR-125b as potential useful marker to predict successful outcome of rituximab treatment. This is the first time a miRNA is identified as potential biomarker for treatment efficacy and prediction of individual targeted therapy in RA.

## Figures and Tables

**Table 1:** RA patient characteristics.

	Blood analysis	Sera analysis			
	RA patients (*n* = 16)	Responders (*n* = 16)		Nonresponders (*n* = 16)	
	Mean ± s.d.	Mean ± s.d.		Mean ± s.d.	
Age (years)	59.1 ± 3	59.8 ± 3		57.6 ± 15	
Gender (%F)	87.5	80.8		85.7	
Disease duration (years)	16 ± 2.2	13.7 ± 1.45		16.3 ± 2.6	
ACPA positive (%)	81.3	87.5		78.6	
DMARDs failed	3.5 ± 0.25	3.7 ± 0.25		3.5 ± 0.4	
		**M0**	**M3**	**M0**	**M3**
Baseline DAS 28	5.4 ± 0.25	6.2 ± 0.2	3.7 ± 0.2	4.8 ± 0.3	5.2 ± 0.3
Baseline CRP level (mg/L)	12.3 ± 4	29.4 ± 6	11.4 ± 4	20.2 ± 8	15.3 ± 5
Baseline HAQ level	1.4 ± 0.1	1.7 ± 0.1	1.4 ± 0.2	1.75 ± 0.2	1.5 ± 0.2

DAS 28 score: a measure of RA activity score (28 joints were evaluated); DMARDs: disease modifying antirheumatic drugs; s.d.: standard deviation; ACPA: anticitrullinated protein/peptide antibodies; CRP: C-reactive protein l; HAQ: health assessment questionnaire.

**Table 2:** B lymphoma patient characteristics.

Sera analysis		
	Responders (*n* = 8)	Nonresponders (*n* = 5)
Median age (years)	56 (52–79)	59
Gender (M/F)	7/1	2/3
Histology		
Diffuse large B cells	7	1
Indolent	1	4
Stage		
I/II	3	2
III/IV	5	3
Treatment		
Rituximab monotherapy	5	1
Rituximab chemotherapy	3	4
